# Supplementary material for: Wafer-scale integration of sacrificial nanofluidic chips for detecting and manipulating single DNA molecules
Source: Nat Commun. 2017 Jan 23;8:14243. doi: 10.1038/ncomms14243 (PMC5264239; doi:10.1038/ncomms14243)
Supplement: Supplementary Information — Supplementary Figures 1-14, Supplementary Notes 1-6 and Supplementary References [file ncomms14243-s1.pdf]

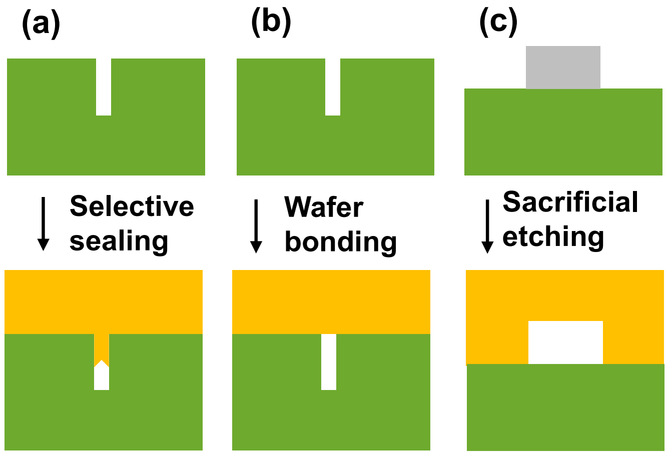

Supplementary Figure 1. Conventional nanofluidic sealing process. (a) Selective sealing. (b) Wafer bonding. (c) Sacrificial etching.

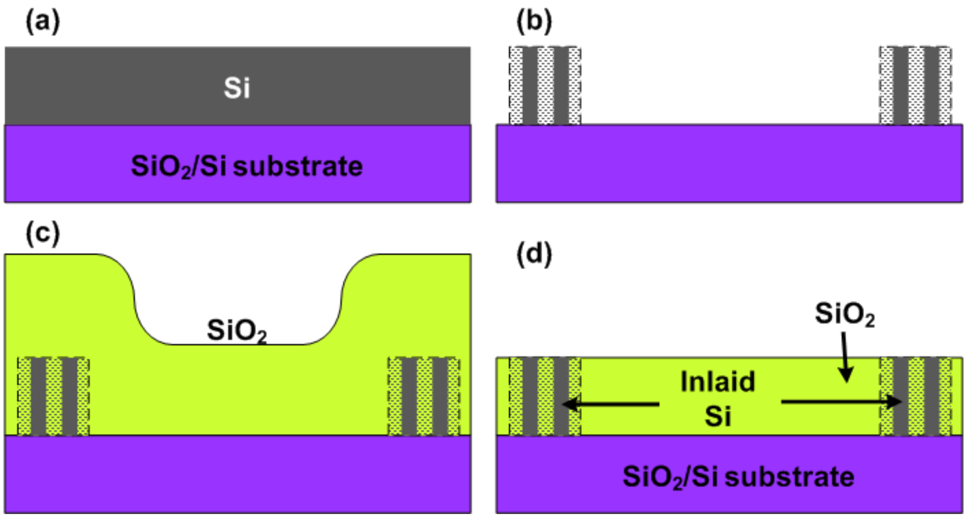

Supplementary Figure 2. Process schematics for interlaying Si microstructures (cross-sectional view). (a) Sacrificial Si layer on SiO_2/Si substrates. (b) Micro-patterned α-Si layer on SiO_2/Si. (c) PECVD SiO_2 deposited on micro-patterned α-Si. (d) Si microstructures inlaid in SiO_2. The Si microstructures were filled in solid and dots to illustrate the two-dimensional patterns.

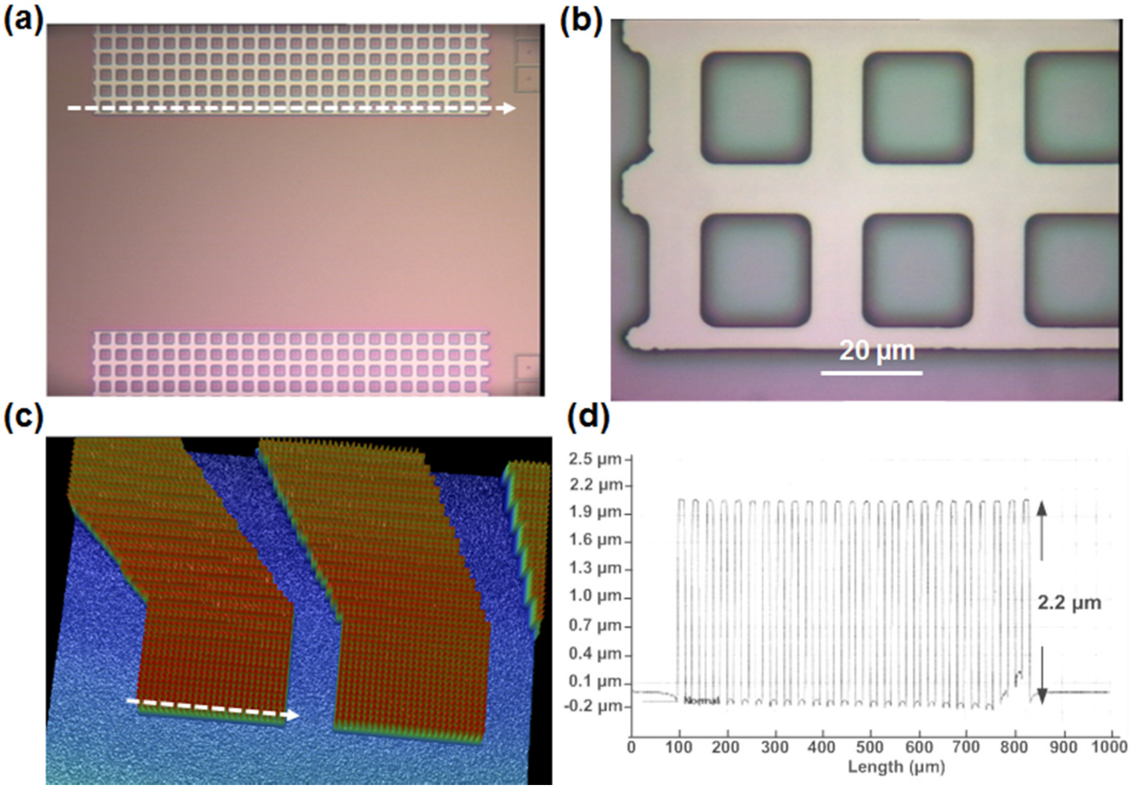

Supplementary Figure 3. Patterned α-Si microchannel structures on SiO_2. (a) Optical image showing the top and bottom branches of the α-Si microstructures. (b) The zoon-in image showing the mesh-like α-Si microstructures. (c) Interferometry measured surface profile. (d) Measured surface step height of 2.2 μm along the α-Si structures, as indicated by arrows in a and c.

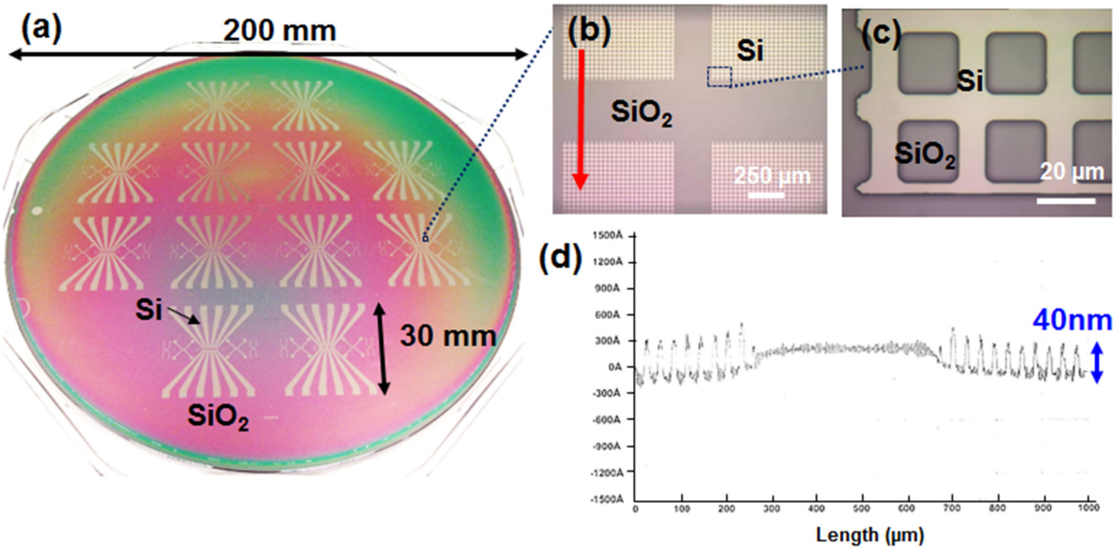

Supplementary Figure 4. Planarized α-Si microstructures inlaid in SiO_2. (a) Optical image of a 200 mm wafer after polishing. (b-c) Optical image of microstructures after polishing. (d) Measured surface step height of 40 nm. The red arrow indicates the scanning direction in surface measurement.

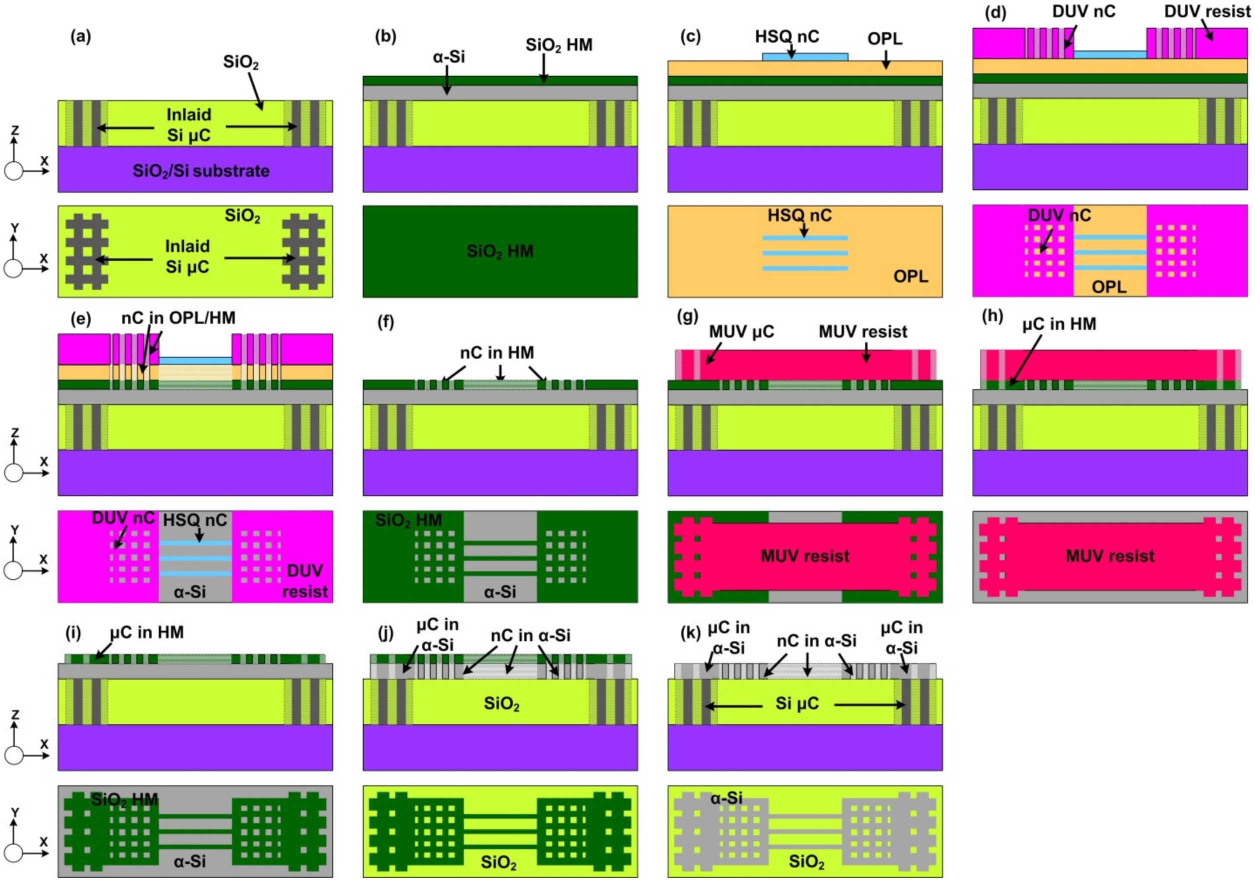

Supplementary Figure 5. Detailed fabrication scheme of patterning sacrificial nanostructures. (a) Sacrificial Si microchannel (μC) structures inlaid in SiO_2 after CMP. (b) Deposition of thin-film SiO_2 hard-mask (HM) and α-Si layers on sacrificial Si microstructures. (c) Deposition of an organic planarization layer (OPL) and a thin HSQ EBL resist, followed by EBL exposure and development to form nanochannels (nC) patterns in HSQ. (d) Nanopatterning of nanofluidic structures in DUV resist (DUV-nC) aligned to HSQ nC. (e) Plasma etching transfer HSQ nC and DUV nC patterns to OPL and HM layers. (f) Stripping the DUV resist, HSQ, and OPL layers by RIE and wet cleaning. (g) MUV lithography to pattern μC structures in MUV resist aligned to underlying inlaid Si μC structures and DUV/EBL nanostructures in HM. (h) Plasma etching to transfer μC structures to HM layer. (i) Stripping MUV resist. (j) Plasma etching to transfer nc and μC features from HM layer to α-Si layer. (k) (Optional) Stripping HM layer. The cross-sectional view (X-Z plane) is shown above, and the top view (X-Y plane) is shown at the bottom in each figure. The solid-filled regions in the resist, HM, and Si layers indicate the materials are not patterned, and the line- and dot-filled regions in cross-sectional view indicated the materials are patterned in two-dimensional fashion.

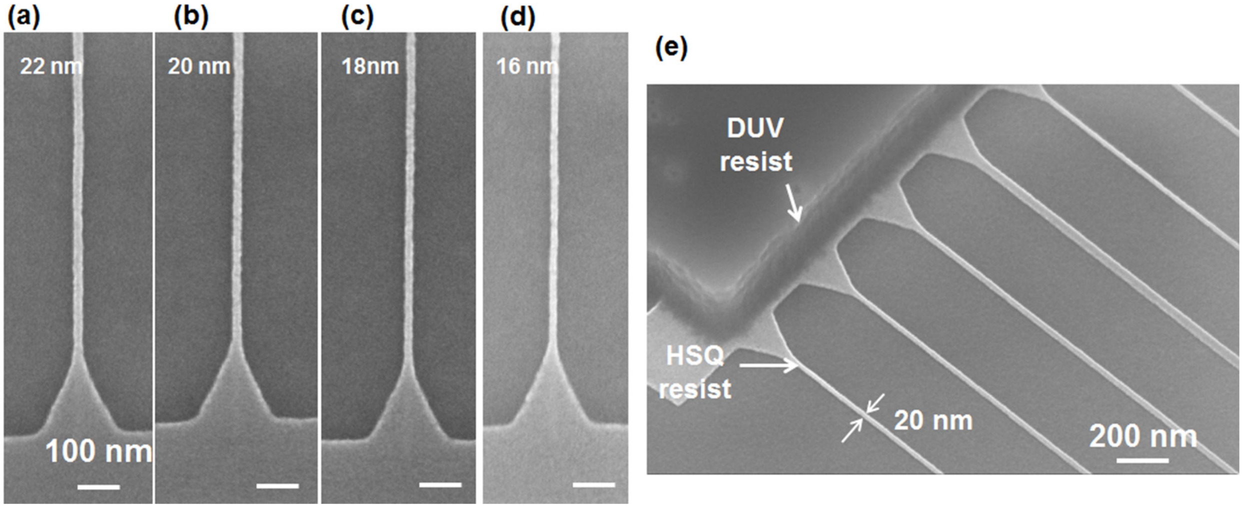

Supplementary Figure 6. Nanofeatures patterned by EBL. (a-d) Nanostructure feature dimensions are carefully tested before implementing wafer-scale fabrication. The dose is from 2400 μC/cm^2 for figure a to 1800 μC/cm^2 for figure d. (e) Tilted SEM image showing well aligned DUV and EBL nanostructures.

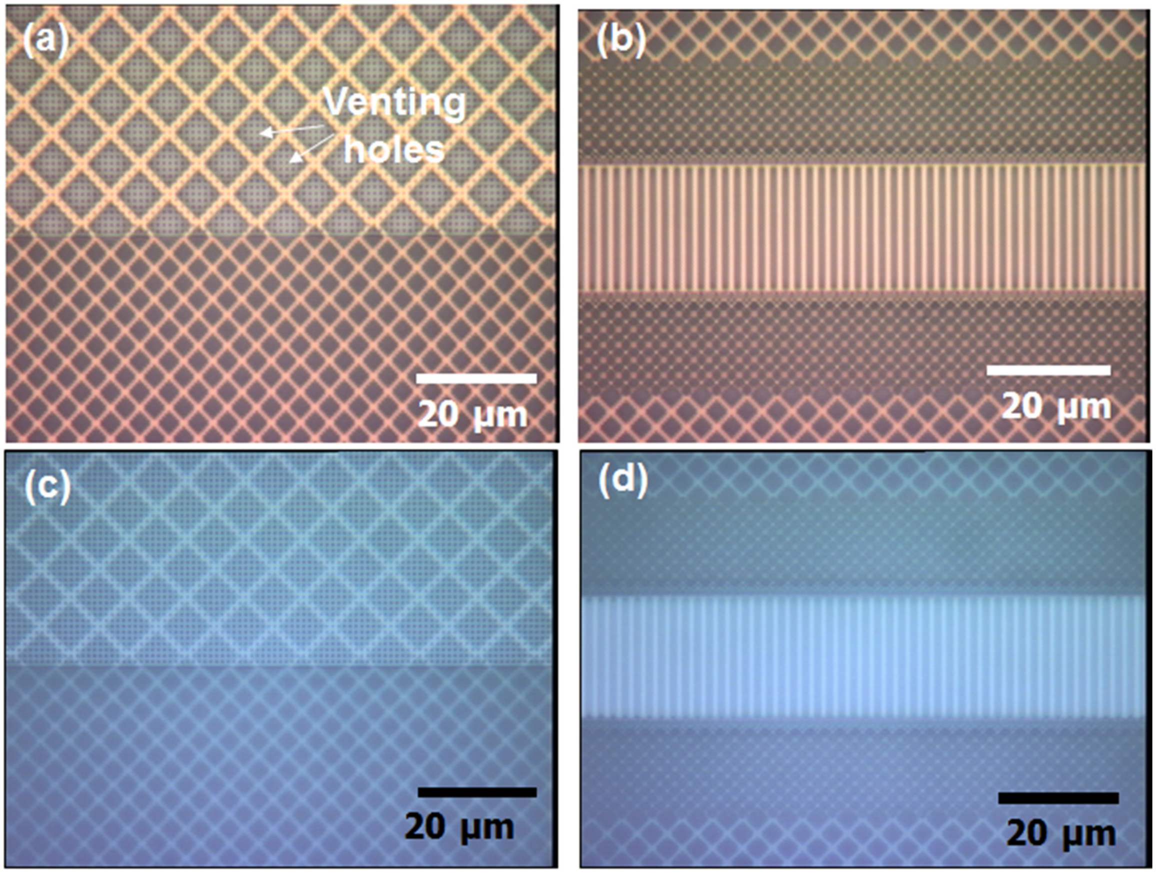

Supplementary Figure 7. Si extraction by XeF_2 etching: a-b, before etching; c-d, after etching. The venting nanoholes are indicated by arrows.

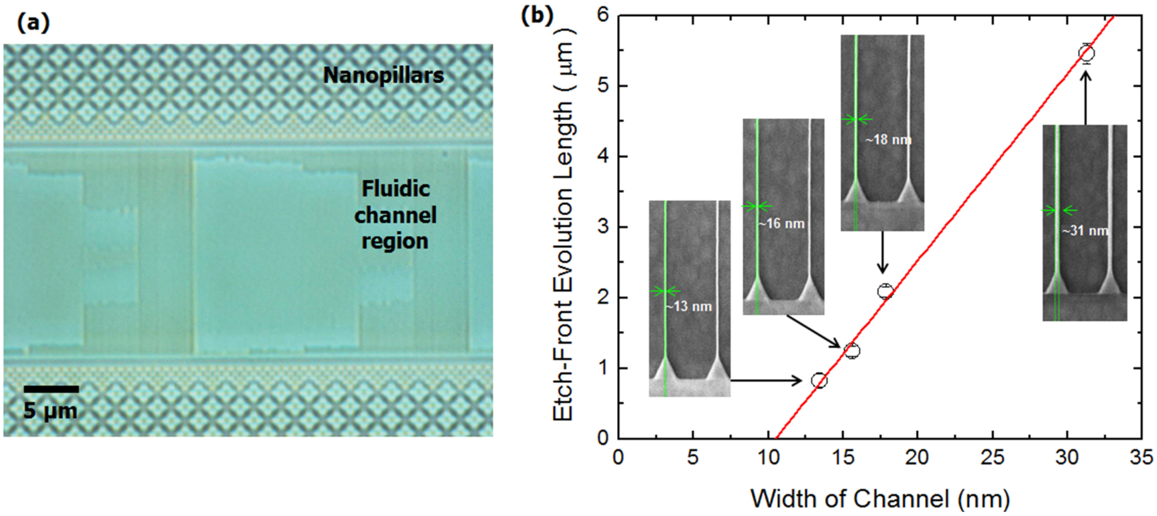

Supplementary Figure 8. Dimension-dependent Si extraction process in nanochannels. (a) An optical microscope image showing the evolution of etch-front of the Si nanochannels during XeF_2 etching process. The widths of the nanochannels are 13 nm, 16 nm, 18 nm, 31 nm and 67 nm. The Si thickness is 40 nm. (b) The etch front plotted against the channel-widths (right), showing the feasibility of XeF_2 Si etching at sub-20 nm.

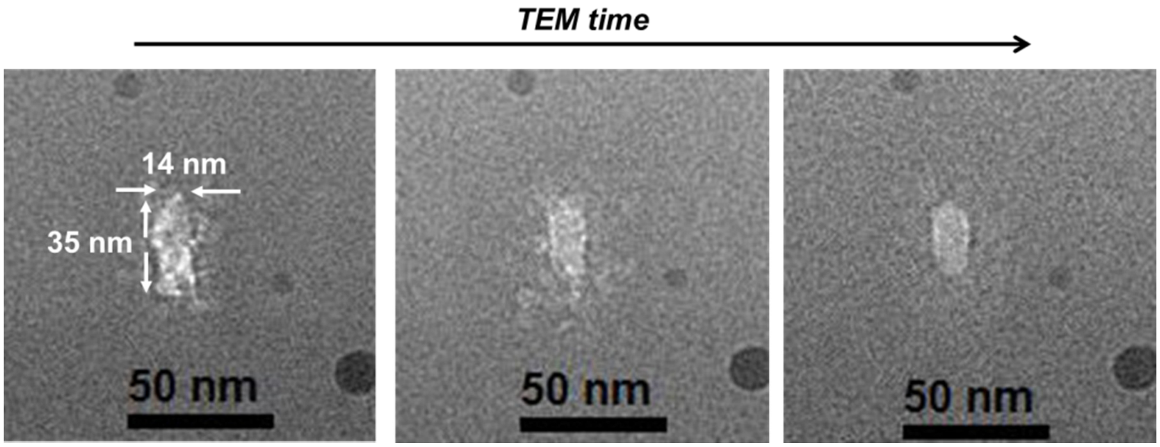

Supplementary Figure 9. Nanochannel closure under TEM observation.

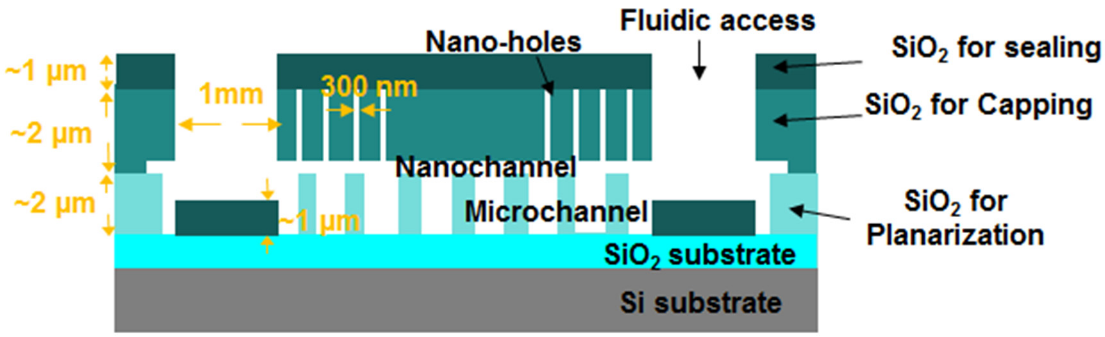

Supplementary Figure 10. Cross-sectional views showing deposited SiO_2 materials involved in nanofluidic venting and sealing. Dimensions are not to scale.

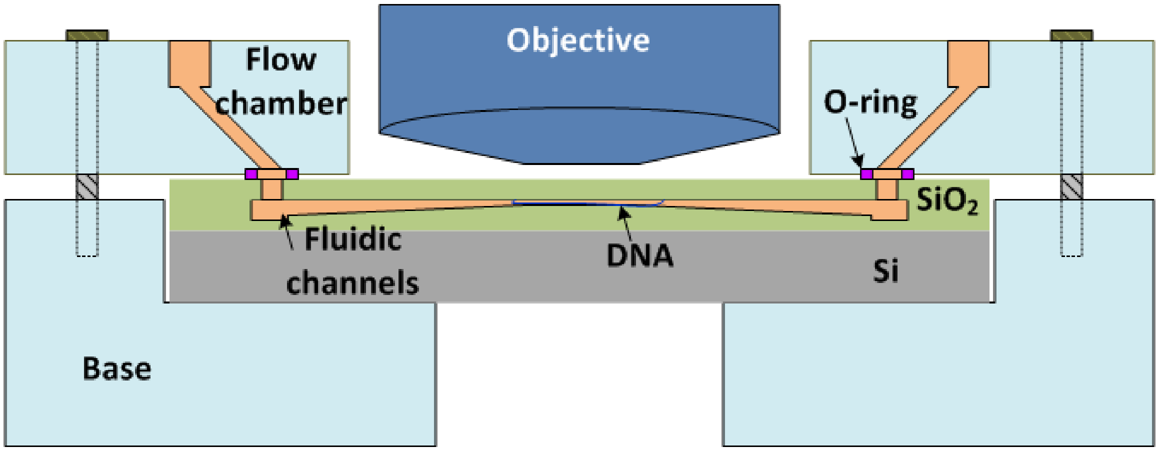

Supplementary Figure 11. Schematic showing the setup for DNA fluorescence imaging in sacrificial nanofluidic devices.

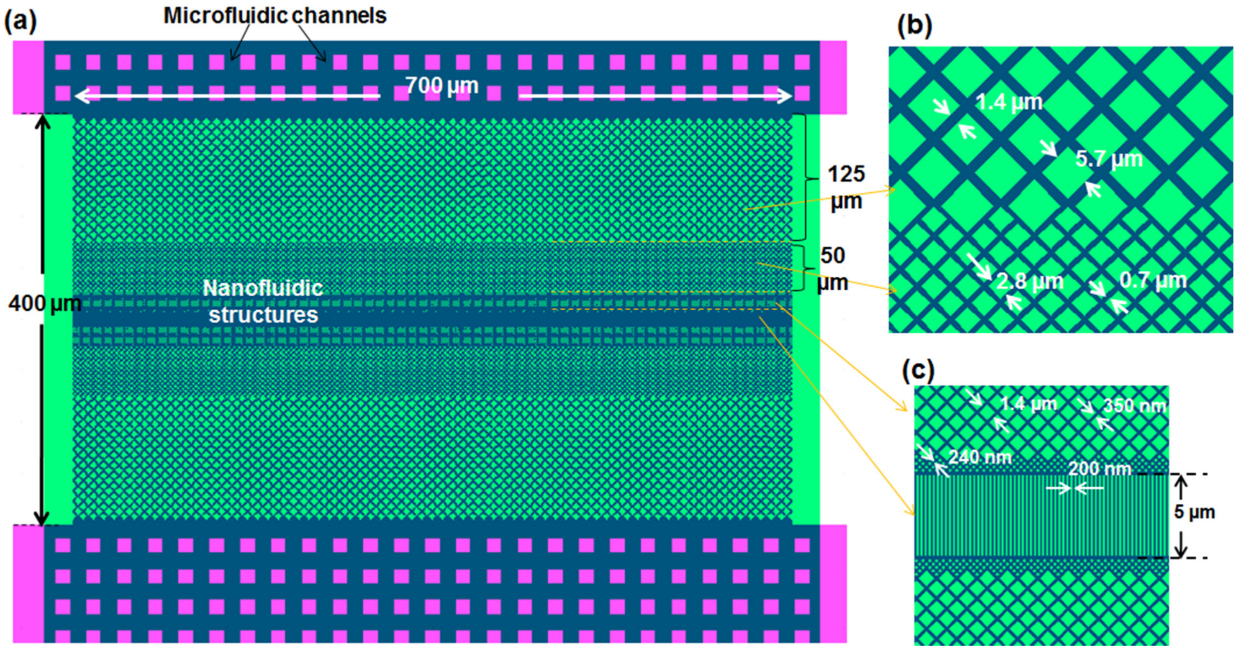

Supplementary Figure 12. Nanofluidic structure design for DNA straddling demonstration. (a) The overall fluidic structure design of one fluidic branch (700 µm wide), with the nanofluidic structures (magenta) printed by DUV lithography and the connecting microfluidic structures (green) printed by MUV lithography. Note the sacrificial Si fluidic structures are those overlapped by the two layers (blue color), please refer to Supplementary Figure 5 for integration details (EBL is replaced by DUV for this design). (b-c) Schematics showing detailed nanofluidic structure dimensions.

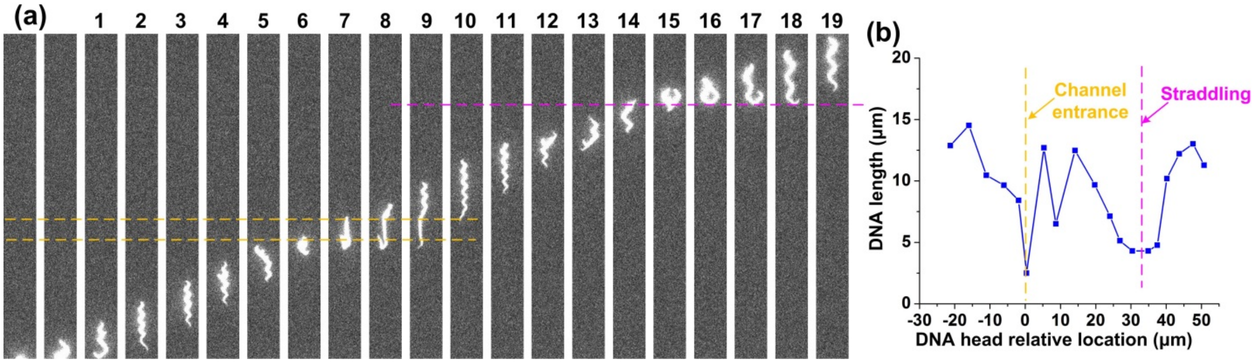

Supplementary Figure 13. Single-molecule DNA fluorescence imaging and analysis. (a) Representative fluorescence images showing λ-DNA flowing through nanopillars and nanochannels. Yellow dash lines indicate the nanochannels, and magenta dash line indicates the pillar interface for straddling. Here frame 1 is defined the first frame the DNA molecule enters the imaged area. The DNA flowed from the bottom to the top. (b) The location-dependent DNA extension due to its hydrodynamic interactions with nanostructures. Here the origin of the DNA location is set as the nanochannel entry. The time interval between adjacent frames was about 18 msec.

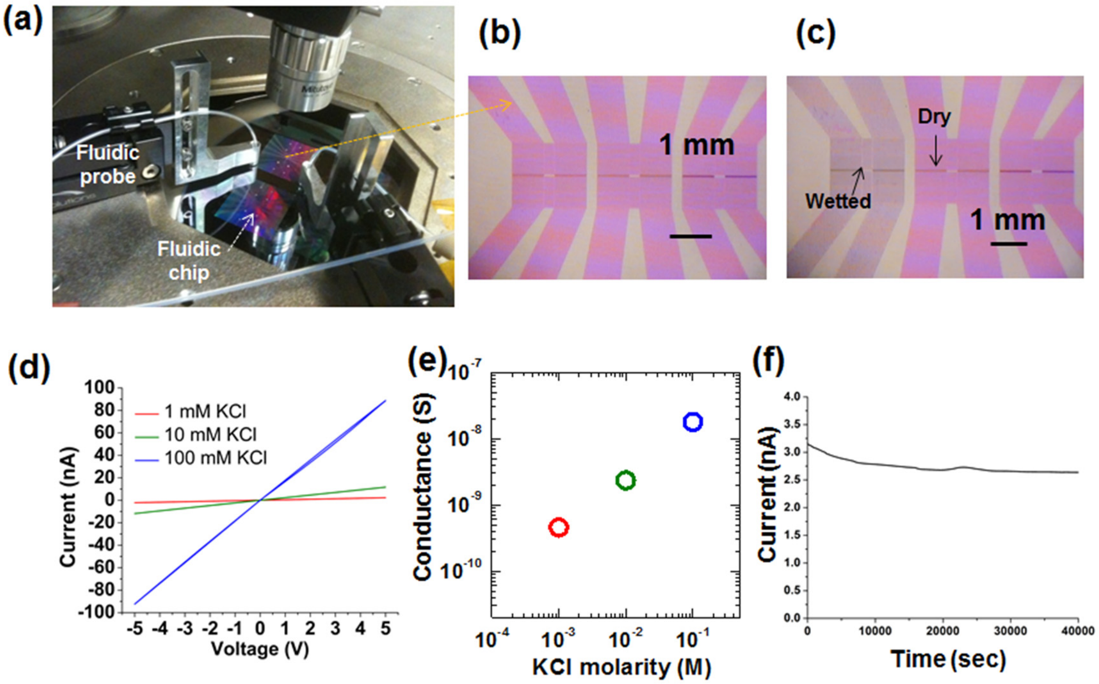

Supplementary Figure 14. Electrical conductivity measurement setup of fluidic channels. (a) Optical image of fluidic probe connected to a nanofluidic chip on an automated probe station. (b, c) Optical images of fluidic chips before and after wetting one fluidic branch (left most branch). (d,e) IV measurement of a fluidic channel branch at different KCl molarity (1 mM, 10 mM, and 100 mM). (f) The ionic current measured across the fluidic access holes over >11.1 hour (40,000 sec) at 5 V bias.

Supplementary Note 1: Conventional nanofluidic manufacturing methods

Unlike conventional nanopores where the fluid sealing is achieved by packaging, planar nanofluidic structures must incorporate a reliable sealing mechanism in the manufacturing process in order to control the fluidic flow. However, existing manufacturing technologies, which generally exploit selective sealing, wafer bonding, or sacrificial materials to create an enclosed nanofluidic system (Supplementary Figure 1), cannot meet all the requirements of patterning sub-5 nm features, producing complex planar fluidic structures, and also integrating metallic sensors over a wafer scale.

Selective sealing methods (Supplementary Figure 1a) utilize dielectric deposition[1] or radiation induced melting[2] to form a continuous film covering the nanochannels surface but leave voids underneath the film for fluid transport. However, these methods demand special materials and nanostructure geometries (*e.g.,* height, width, shape, *etc.*), and thus cannot be applied universally. In the case of the wafer bonding (Supplementary Figure 1b), nanopatterned substrates are sealed by a second substrate. Bonding to rigid materials[3,4] followed by a high-temperature (usually 300 to 1000°C) annealing can yield a strong bonding strength, suitable for applications requiring a precise control of structural dimensions. However, the stringent requirements of bonding surface cleanliness and high-temperature annealing process pose yield and metal integration challenges. Although bonding to soft materials (elastomers and polymers[5,6]) can partially alleviate these difficulties, it has many drawbacks such as leakage, low bonding strength, clogging due to polymer deformation, and incompatibility with various chemicals.

In comparison, sacrificial approaches (Supplementary Figure 1c) offers a promise of creating complex nanofluidic structures with embedded functional CMOS electronic components. Such approaches utilize a material "to be sacrificed", either organic polymers[7] or inorganic materials such as Si [8,9] and SiO_2[10], patterned into a reverse image of the desired nanofluidic structures (*e.g.* isolated pillars versus meshes), and selectively extract this sacrificial material at a later stage of processing to form the nanofluidic system by thermal decomposition[7] or wet chemical etching[8-10]. However, thermal decomposition has serious risks of structural damage at elevated temperatures. Wet etching processes are ineffective at nanometer scales and potentially destructive to the nanofluidic structures, because removing etched byproduct becomes exceedingly difficult and undesirable long processing time is needed (*e.g.* >40-80 hours for millimeter long channels at micron scales)[9].

Supplementary Note 2: Substrate planarization

The key steps to inlay sacrificial Si microstructures include deposition of a sacrificial amorphous Si (α-Si) layer (~2 μm thick) (Supplementary Figure 2 a), α-Si micropatterning by mid-UV contact lithography (MUV) and plasma etch (Supplementary Figure 2 b), SiO_2 conformal coating by plasma-enhanced chemical vapor deposition (PECVD) (Supplementary

Figure 2 c), and wafer surface polishing by chemical mechanical polishing (CMP) (Supplementary Figure 2 d). The fabricated fluidic microstructures are shown in Supplementary Figure 3. The step height was measured to be 2.2 µm before polishing (Supplementary Figure 3) and was reduced to 40 nm after polishing (Supplementary Figure 4).

Supplementary Note 3: Sacrificial Si nanostructure patterning

In this work, we utilize established procedures and recipes at IBM MRL lab during critical nanopatterning steps to maximize the feature uniformity and yield. For example, the plasma etch uniformity in our etch chamber has an etch rate uniformity of within 5% across a 200mm wafer and from wafer-to-wafer. The critical dimension (CD) in DUV lithography has a <15nm variation for a 200nm line/space standard design across a 200 mm wafer, and the yield is about 100% for the dimensions in this work (critical dimension ~200 nm). The high yield is achieved by printing in a controlled and fully automated environment of an ASML and TEL track without manual handling and by applying internal stepper diagnostics on a regular basis to control the focus and dose. To optimize the alignment accuracy between the EBL and DUV fluidic nanostructures, we patterned alignment marks on the substrate prior to nanopatterning, consisting of marks designed for EBL and DUV. We used the same alignment mark sets in both EBL and DUV nanopatterning, hence minimizing the alignment errors. Taking into account the alignment accuracies of different levels, *i.e.* <20 nm for EBL and DUV and about 1 µm for MUV, we carefully designed the corresponding fluidic structures with large enough tolerance.

The detailed fabrication of the nanofluidic structures (Supplementary Figure 5) are given in Methods section. Prior to wafer-scale fabrication, we carefully examined the dimensions of the nanochannels over the 8 inch wafer. For example, by changing the electron-dose from 2400 uC/cm^2 to 1800 uC/cm^2 in EBL process, the pattern dimensions could be tuned from 22 nm to 16 nm in hydrogen-silsesquioxane (HSQ) (Supplementary Figure 6 a-d). In addition, the accurate alignment between the patterns of EBL-patterned HSQ resist and DUV resist (Supplementary Figure 6 e) is critical to producing a continuous sacrificial Si layer incorporating both nano- and micro-structures.

Supplementary Note 4: Sacrificial Si extraction

The high optical contrast between Si and SiO_2 allows us to conveniently monitor the XeF_2 etching process of Si (Supplementary Figure 7). In our layout-design, the venting holes are separated by 60 µm (Figure 3). The sacrificial Si materials in the nanochannels did not have any venting holes patterned on the top; instead, they are extracted through the venting holes patterned on top of the micrometer- and nanometer-sized channels connecting the critical nanochannels. To investigate the effect of the feature size on the XeF_2 etching process, we monitored the location of the Si etch-front by optical microscopy (Supplementary Figure 8 a). In the

experiments, we tested the feature size from ~70 nm down to ~13 nm. Clearly, the XeF_2 etching rate of amorphous Si in wider nanochannels was much faster than narrower channels (Supplementary Figure 8 b).

The size-dependent Si etching rate can be understood as a result of size-dependent vapor-phase transport of the XeF_2 precursor to Si surface and the volatile byproducts away from the Si surface. Obviously, the diffusion of XeF_2 gas and by-product is slower within narrower channels. This can be attributed to higher probability of gas molecules to collide with the nanochannels sidewalls at vacuum (3 Torr XeF_2, 15 Torr N_2 in our experiment), in agreement with Knudsen diffusion model. Our experimental results also showed a linear dependence of etching rate versus channel dimensions, probably because the diffusivity is proportional to the critical dimensions of the nanochannels at the Knudsen diffusion regime. In spite of the slow etch-rate in the narrow (sub-20 nm) nanochannels, the successful etching can be completed by increasing XeF_2 gas-purging time and cycles. In our experiments, 20 μm long, sub-20 nm wide, and 40 nm high nanochannels were successfully extracted.

The very narrow nanochannels were found to close under TEM observation. For example, an initially measured 14 nm wide channel were observed to close during TEM imaging (Supplementary Figure 9). Although not fully understood at this stage, this effect is probably attributed to the electron beam induced carbon deposition at the nanochannels and/or melting and coalescence of PECVD SiO_2 under high-energy electron beam irradiation (possibly driven by surface energy).

Supplementary Figure 10 summarizes the SiO_2 materials used in the sacrificial Si patterning, extraction, and sealing processes. SiO_2 is deposited for three different purposes – substrate planarization, Si structure capping, and venting hole sealing. The PECVD SiO_2 substrate and the planarization, capping, and sealing SiO_2 films define the eventual microfluidic and nanofluidic device structures.

Supplementary Note 5: Nanofluidic structures and DNA hydrodynamic studies

Here we aim at demonstrating the capabilities of our sacrificial Si strategy of integrating complex and functional nanofluidic structures using a customized fluidic jig (Supplementary Figure 11). In our design, the nanopillar design is the key to achieving complex DNA hydrodynamic interactions, and the nanofluidic channel dimensions are not critical. The devices were fabricated following the strategy we detailed in previous section (Supplementary Figure 5), but here we chose DUV lithography rather than EBL to fabricate the nanofluidic structures (Supplementary Figure 12), similar to our previous report.[4] Each fluidic chip was designed to have six isolated fluidic branches, which have identical pillar designs. Within each fluidic branch (Supplementary Figure 12 a), the nanofluidic pillars and channels were patterned in an area of 700 μm × 400 μm and connected by microchannels on both sides. The nanofluidic structures

included nanochannels in the middle surrounded by symmetrically arranged diamond-shaped nanopillars on each side (Supplementary Figure 12 b-c). The fluidic design featured diamond-shaped nanopillars with abruptly designed interface to control DNA straddling interaction[11] and pillar gaps that are progressively reduced in dimensions from 1.4 μm to 240 nm, functioning effectively as cascaded two-dimensional fluidic network to pre-stretch the DNA.[3]

With consecutively captured fluorescence images (exposure time 17.8 ms, frame cycle time 18.1 ms), we studied the single-molecule λ-DNA molecule translocation through the nanopillar and nanochannel regions (Figure 5). The frame-by-frame speeds and extensions of these DNA molecules were derived by measuring the DNA head and tail locations. The extension L is the fluorescently measured length L_M corrected by DNA travel distance during exposure time τ using measured DNA frame speed v through the relation $L = L_M - v\tau$. The average DNA speed in the imaged region is $140\ \mu m/sec$. Clearly from Figure 5, the extensions of DNA molecules are strongly correlated to the nanofluidic structure design. In this report, we do not focus on the detailed DNA hydrodynamic interactions with the diamond-shaped pillars, which have thoroughly analyzed in our previous report using similar structures.[4]

In a different chip, the DNA molecules have similar hydrodynamic flow, straddling, and relaxation interactions with the nanopillars (Supplementary Figure 13). Clearly, the DNA molecule stretched much longer after entering the nanochannels (Supplementary Figure 13 a, frames 6-10) and also straddling nanopillars (Supplementary Figure 13 a, frames 14-18). This demonstration further illustrates the complexity of DNA hydrodynamic behaviors in nanofluidic structures, and also emphasizes the importance of our integration strategy in nanofluidics and single molecule studies.

Supplementary Note 6: Fluidic chip electrical test

The aim of this work is to demonstrate the feasibility of our strategy in wafer-scale integration of complex nanochannels and compatibility with single molecule fluorescence imaging. In this work, we also carried out ionic conductance measurement using our fluidic probe stations on two randomly selected chips at the edges of the 200 mm wafer. To evaluate the fluidic connection, we used a fluidic probe (Qmix, CETONI GmbH, Germany) to deliver a KCl buffer solution to the fluidic chip and simultaneously control the pressure and flow rate (Figure S14 a). An optical microscope with a CCD camera was mounted onto the probe station to visualize the fluidic channel regions during test (Supplementary Figure 14 b-c). The fluidic chips were first wetted by DI water under a typical pressure of 0.5 Bar. No leakage was observed using a pressure as high as 11 Bar, and no higher pressure was attempted. Then, the DI water was replaced by KCl solutions (pH 5.5) at different molarities, *i.e.* 1 mM to 100 mM. Using Ag/AgCl wires inserted in the fluidic probes in contact with the KCl buffer, we measured the electrical conductance of the fluidic chip across two fluidic access ports as 0.44, 2.3, 17.7 nS, respectively. The linear

dependence of conductance on salt molarity indicated a complete wetting of the fluidic chips. In addition, the ionic current of the fluidic chips was stable over 11.1 hours (Supplementary Figure 14 f), indicating good device stability. From two wetted fluidic branches on each of the two randomly selected chips, we obtained very similar ionic current (variation <10%). The good agreement is attributed to a few reasons. First, the nanofluidic structures have uniform dimensions. Second, the nanofluidic channels are fully wet. Thirdly, the two-dimensional fluidic network in our design has many parallel channels connecting the inlet and outlet, and hence has a much more stable current compared to a single channel. The small variation is attributed to occasional air bubbles injected by the fluidic probes, interface resistance at the Ag/AgCl electrodes, etc.

Supplementary References:

1 Cao, H. *et al.* Fabrication of 10 nm enclosed nanofluidic channels. *Appl. Phys. Lett.* **81**, 174-176, (2002).

2 Xia, Q. F., Morton, K. J., Austin, R. H. & Chou, S. Y. Sub-10 nm Self-Enclosed Self-Limited Nanofluidic Channel Arrays. *Nano Lett.* **8**, 3830-3833, (2008).

3 Reisner, W. *et al.* Statics and dynamics of single DNA molecules confined in nanochannels. *Phys. Rev. Lett.* **94**, 196101, (2005).

4 Wang, C. *et al.* Hydrodynamics of diamond-shaped gradient nanopillar arrays for effective DNA translocation into nanochannels. *ACS Nano* **9**, 1206-1218, (2015).

5 Liang, X. G. & Chou, S. Y. Nanogap detector inside nanofluidic channel for fast real-time label-free DNA analysis. *Nano Lett.* **8**, 1472-1476, (2008).

6 Becker, H. & Gärtner, C. Polymer microfabrication technologies for microfluidic systems. *Anal. Bioanal. Chem.* **390**, 89-111, (2008).

7 Li, W. *et al.* Sacrificial polymers for nanofluidic channels in biological applications. *Nanotechnology* **14**, 578, (2003).

8 Turner, S., Perez, A., Lopez, A. & Craighead, H. Monolithic nanofluid sieving structures for DNA manipulation. *J. Vac. Sci. Technol., B* **16**, 3835-3840, (1998).

9 Stern, M. B., Geis, M. W. & Curtin, J. E. Nanochannel fabrication for chemical sensors. *J. Vac. Sci. Technol., B* **15**, 2887-2891, (1997).

10 Lee, C., Yang, E.-H., Myung, N. V. & George, T. A nanochannel fabrication technique without nanolithography. *Nano Lett.* **3**, 1339-1340, (2003).

11 Viero, Y., He, Q. H. & Bancaud, A. Hydrodynamic Manipulation of DNA in Nanopost Arrays: Unhooking Dynamics and Size Separation. *Small* **7**, 3508-3518, (2011).
